# Predicting Unprecedented Dengue Outbreak Using Imported Cases and Climatic Factors in Guangzhou, 2014

**DOI:** 10.1371/journal.pntd.0003808

**Published:** 2015-05-28

**Authors:** Shaowei Sang, Shaohua Gu, Peng Bi, Weizhong Yang, Zhicong Yang, Lei Xu, Jun Yang, Xiaobo Liu, Tong Jiang, Haixia Wu, Cordia Chu, Qiyong Liu

**Affiliations:** 1 State Key Laboratory for Infectious Disease Prevention and Control, Collaborative Innovation Center for Diagnosis and Treatment of Infectious Diseases, National Institute for Communicable Disease Control and Prevention, Chinese Center for Disease Control and Prevention, Changping, Beijing, People’s Republic of China; 2 Shandong University Climate Change and Health Center, Jinan, Shandong, People’s Republic of China; 3 School of Population Health, University of Adelaide, Adelaide, South Australia, Australia; 4 Key Laboratory of Surveillance and Early-Warning on Infectious Disease, Division of Infectious Disease, Chinese Center for Disease Control and Prevention, Beijing, People’s Republic of China; 5 Guangzhou Center for Disease Control and Prevention, Guangzhou, People’s Republic of China; 6 National Climate Center, China Meteorological Administration, Beijing, People’s Republic of China; 7 Centre for Environment and Population Health, Nathan Campus, Griffith University, Queensland, Nathan, Australia; Santa Fe Institute, UNITED STATES

## Abstract

**Introduction:**

Dengue is endemic in more than 100 countries, mainly in tropical and subtropical regions, and the incidence has increased 30-fold in the past 50 years. The situation of dengue in China has become more and more severe, with an unprecedented dengue outbreak hitting south China in 2014. Building a dengue early warning system is therefore urgent and necessary for timely and effective response.

**Methodology and Principal Findings:**

In the study we developed a time series Poisson multivariate regression model using imported dengue cases, local minimum temperature and accumulative precipitation to predict the dengue occurrence in four districts of Guangzhou, China. The time series data were decomposed into seasonal, trend and remainder components using a seasonal-trend decomposition procedure based on loess (STL). The time lag of climatic factors included in the model was chosen based on Spearman correlation analysis. Autocorrelation, seasonality and long-term trend were controlled in the model. A best model was selected and validated using Generalized Cross Validation (GCV) score and residual test. The data from March 2006 to December 2012 were used to develop the model while the data from January 2013 to September 2014 were employed to validate the model. Time series Poisson model showed that imported cases in the previous month, minimum temperature in the previous month and accumulative precipitation with three month lags could project the dengue outbreaks occurred in 2013 and 2014 after controlling the autocorrelation, seasonality and long-term trend.

**Conclusions:**

Together with the sole transmission vector *Aedes albopictus*, imported cases, monthly minimum temperature and monthly accumulative precipitation may be used to develop a low-cost effective early warning system.

## Author Summary

Dengue has emerged as the most important mosquito-borne viral disease globally. With increasing global trade and population movement, the disease is transferred to regions which were previously dengue free. When dengue vector exists and weather factors are suitable, there is the possibility for dengue transmission and even outbreaks happening. Dengue is still believed to be a non-endemic disease in China, with imported cases playing a vital role in local dengue transmission. The situation of dengue is becoming more and more severe with two successive large outbreaks hitting southern China in 2013 and 2014, and the dengue outbreak in 2014 was unprecedented. In this study, we aim to develop a dengue forecasting model that would provide an early warning of dengue outbreak to allow local health authorities and communities to implement timely effective control measures. Our model showed that imported cases in the previous month, monthly minimum temperature in the previous month and monthly accumulative precipitation with three month lags could predict dengue outbreak ahead by one month. We concluded that these variables could be used to develop an early dengue warning model to provide evidence-based decisions for disease control and prevention and including the utilization of limited resources.

## Introduction

Dengue is an arthropod-borne disease caused by dengue virus (DENV 1–4) belonging to the *Flaviviridae* family, with the transmission vectors being *Aedes aegypti* and *Aedes albopictus* [1]. Dengue is endemic in more than 100 countries worldwide, mainly in tropical and subtropical regions [2]. Recent global estimates indicate that 390 million people have dengue virus infections with 96 million cases annually [3]. Its incidence has increased 30-fold in the past 50 years [4]. Because of unprecedented population growth, globalisation with increased population movement, uncontrolled urbanisation, climate change, breakdown in public health infrastructure and vector control programs, dengue is the most prevalent and rapidly spreading mosquito-borne viral disease affecting human beings [1].

WHO has defined a global strategy for dengue prevention and control, aimed to reduce mortality and morbidity from dengue at least 50% and 25% by 2020 respectively (using 2010 as the baseline) [4]. Evidence-based decisions are essential to prevent and control dengue transmission. A dengue early warning system will be helpful to provide evidence for decision-makers.

Human movement has been identified as one of key factors in determining the transmission dynamics of dengue disease [5]. Movements into high-risk areas lead to individual infection, and also contribute to local transmission when infected individuals return to their homes where local transmission vectors establish. Madeira, Portugal reported the first major outbreak of dengue in 2012, which was probably caused by the virus imported from Venezuela [6]. Yunnan Province of China, bordering Cambodia, Thailand and Vietnam reported the first dengue outbreak in 2013 [7] and in the same year the first dengue outbreak occurred in Henan Province located in the central China [8]. Because of population movement and establishment of *Aedes albopictus*, local dengue transmission occurred in France twice in 2010 and 2013, respectively [9,10].

Many researches have studied the association between climatic factors and dengue incidence. Among the climatic factors, temperature and precipitation contributed the most in statistical models [11]. Temperature and precipitation can influence dengue transmission via their impact on the vector population, directly and indirectly [12]. Temperature can impact vector population development and reproductive rates [13]. It is also critical to vector capacity: increased temperature decreases the extrinsic incubation period (EIP), the time taken for mosquitoes from imbibing an infectious blood meal to becoming infectious [14]. It may also affect human behaviour [12]. Precipitation can provide breeding sites and stimulate egg hatching, which leads to an increase in the number of mosquitoes [15]. Based on the relationship between these climatic factors and dengue occurrence, temperature and precipitation were often used to predict or project dengue transmission [16,17].

Since the first dengue outbreak occurred in 1978, dengue has been detected in China for nearly 40 years. Local dengue transmission has been identified in Guangdong, Guangxi, Hainan, Yunnan, Fujian, Zhejiang, and Henan Provinces [8,18]. Two consecutive large outbreaks occurred in Southern China in 2013 and 2014, of which Guangdong Province has had an unprecedented dengue outbreak including 21,511 notifiable cases and six fatalities (up to September 2014, the cases from China Notifiable Reporting System) in 2014. Guangdong Province is located in South-eastern China, and has a subtropical monsoonal climate. The population exchange between Guangdong Province and Southeast Asia is very frequent [19]. Several dengue outbreak occurrences in Guangdong Province were caused by imported cases from Southeast Asia [19,20,21]. Several studies have been conducted to identify the relationship between climatic factors and dengue transmission in Guangdong, yet no real predicting model has been developed [22,23,24]. Given there is no medication which could effectively treat dengue patients [25] and no multivalent dengue vaccines available, preventative measures are crucial in the disease control. An early warning system, based on existing variables, is the backbone of prevention of local cases and possible outbreaks occurring. In this study, we use imported cases and climatic factors to build a low-cost early warning system, capable of predicting dengue outbreak to enhance decision-making capacity.

## Materials and Methods

### Ethics statement

Ethical approval for this project was obtained from the Chinese Center for Disease Control and Prevention Ethical Review Committee (No. 201214) and patient data used in the study were de-identified.

### Study areas

**Fig 1:**
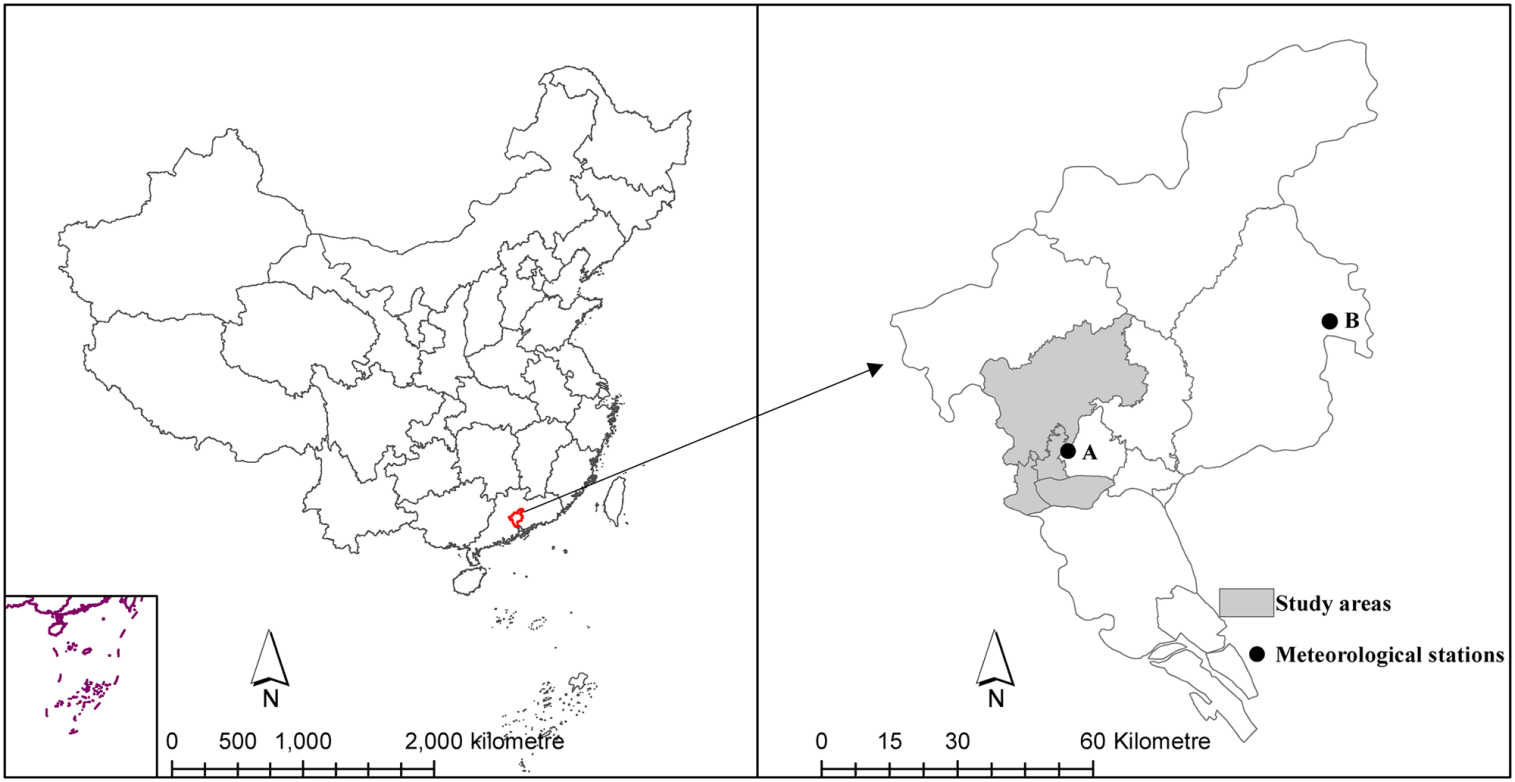
The study areas in Guangzhou city, China.

Guangzhou city is the capital of Guangdong Province, with the highest population density in southern China. Guangzhou is the centre for transportation, industry, finance and trade in southern China and has a large demographic exchange in business, tourism and labour service within Southeast Asia, Africa and the Indian subcontinent. Guangzhou city consists of 12 districts/counties. Given most dengue cases located in Baiyun district, Yuexiu district, Liwan district and Haizhu district, accounted for 74.4% of all cases reported in the 12 districts, we chose the four districts as our study areas, with an area of 979.09 km^2^ and population of 5.81 million in 2013 (Fig 1).

### Data collection

Records of dengue cases between 2006 and 2014 were obtained from the China Notifiable Disease Surveillance System. All dengue cases were diagnosed according to the China National Diagnostic Criteria for dengue (WS216-2008) [26]. Information of dengue cases included age, gender, occupation, date of onset, whether the diagnosis was clinical or confirmed by laboratory test, local case or not. The criteria of imported or local cases used in [12] was cited.

Monthly weather data between March 2006 and September 2014 were obtained from the China Meteorological Data Sharing Service System (http://cdc.nmic.cn/home.do), including monthly minimum temperature, monthly accumulative precipitation. There are two meteorological stations in Guangzhou city (Fig 1), and the climate dataset used was monitored by meteorological station A which is able to represent the weather situation in the study areas. The population data over the study period for every district were retrieved from the Guangdong Statistical Yearbook.

### Statistical analysis

#### Seasonal-trend decomposition procedure based on loess

Seasonal-trend decomposition procedure based on loess (STL) was used to decompose a time series into seasonal, trend, and remainder components using loess [27]. The time series data, the seasonal component, the trend component and the remainder component were denoted by *Y*_t,_ *S*_t,_ *T*_t,_ *R*_t_ respectively, for t = 1 to N. Then

$$Y_{t}\;=\;S_{t}+T_{t}+R_{t}$$

The parameter “periodic” was used on the seasonal extraction, and the other parameters were by default. In the study, *Y*_*t*_ specifically stands for local dengue cases with logarithm transformation, monthly imported cases, monthly minimum temperature, monthly accumulative precipitation with logarithm transformation; *t* is time in unit of month.

#### Time series Poisson regression model construction

Initially, Spearman correlation analysis was applied to identify the correlation between dengue occurrence and minimum temperature and accumulative precipitation with 6 month lags. The correlation results were presented in S1 Table.

We established a time series Poisson regression model to identify the relationship between climatic factors and local dengue incidence. Because the climatic variables with different time lags were highly correlated, only minimum temperature and accumulative precipitation with some month lag based on the spearman correlation result were used to construct the model. Model construction was based on the Generalized Cross Validation (GCV) score. Current dengue cases may be influenced by cases in the past. We analysed the period of this influence by autocorrelation function (ACF) and partial autocorrelation function (PACF). It is still characteristic that dengue is an imported disease in China, our previous study showed that imported cases in the previous month influenced the current dengue occurrence [12]. We applied cubic spline function on these risk factors to allow a non-linear exposure and response association between risk factors and dengue occurrence.

We modeled the predictors as smooth cubic spline functions given 3 degrees of freedom (*df*) each [28]. The sensitivity of the trend was tested by setting *df* to be 4, 5, 6. We used *quasi*-Poisson model to allow for over-dispersion of data. A best model was selected and validated using GCV score and residual test. The data from March 2006 to December 2012 were used to develop the model and the data from January 2013 to September 2014 were employed to validate the model.

$$\text{Log}\left(\mu_{t}\right)\;=\;\text{β}0+\text{S}(\text{Log}\left(local_{t-1}),\text{df}\right)+\text{S}\left(MinT_{t-m},\text{df}\right)+\text{S}\left(\text{Log}(Prept_{t-n}),\text{df}\right)+\text{S}\left(Imp_{t-1},\text{df}\right)+\text{year}+\text{month}+\text{offset}\left(\log\left(\text{pop}\right)\right)$$

μ_t_ represents predicted mean dengue cases in month; S(Log(*local*_t-1_),df) denotes cubic spline of autoregressive terms of dengue cases in the previous month with logarithm transformation with corresponding *df*; S(*MinT*_*t-m*,_df) denotes cubic spline of minimum temperature with corresponding *df*; S(Log(*Prept*_t-n_),df) represents cubic spline of accumulative precipitation with logarithm transformation with corresponding *df*; S(*Imp*_*t-1*,_df) denotes cubic spline of imported cases in the previous month with corresponding *df*; year was used to control long-term trend [29]; month conducted with categorical variable was used to control the seasonality [29]; offset(Log(pop)) was accounted for changes in size of the population [16]. All the analyses were performed by R 3.1.1 [30].

## Results

**Fig 2:**
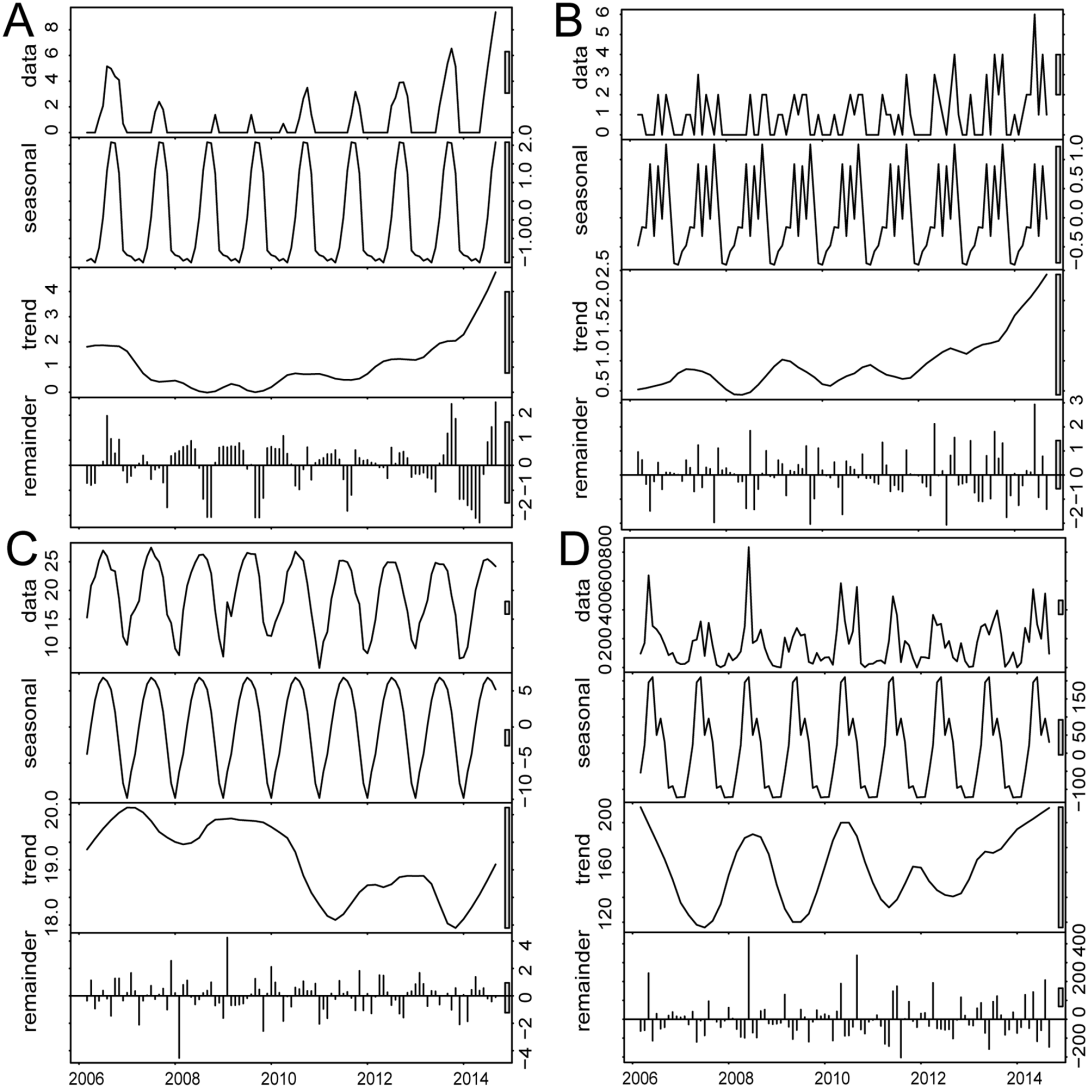
The decomposition plot of the time series in the study areas from March 2006 to September 2014. A) The decomposition plot of local dengue cases with logarithm transformation; B) The decomposition plot of imported cases; C) The decomposition plot of monthly minimum temperature; D) The decomposition plot of monthly accumulative precipitation. The top layer shows the original time series. The other layers show the decomposed components, denoting the seasonal component, long term trend component and remainder component, respectively.

China Notifiable Disease Surveillance System was notified of 15,221 cases, including 15,118 local cases and 103 imported cases between March 2006 and September 2014 in the study areas, with the accumulative local dengue incidence 259 per 100,000. Three large outbreaks occurred in 2006, 2013 and 2014, with the local dengue incidence 9.94, 18.00, and 203.82 per 100,000 respectively. The incidence in 2014 was 3.7 fold than the accumulative incidence from 2006 to 2013. Local dengue occurrence had a seasonal pattern and the epidemic months were from August to November. The decomposition result showed that dengue incidence had an increased trend, especially from 2009 to 2014. Cases imported to Guangzhou also had a seasonal pattern with June, August and October having more imported cases. The result also showed that the number of imported cases had an increased trend pattern. Accumulative precipitation and minimum temperature had seasonal distribution, which were prone to have more precipitation and higher temperature in April-September and May-October, respectively. The accumulative precipitation had an increased trend after 2011, but the minimum temperature had a decreased trend (Fig 2) (S2 Table).

**Fig 3:**
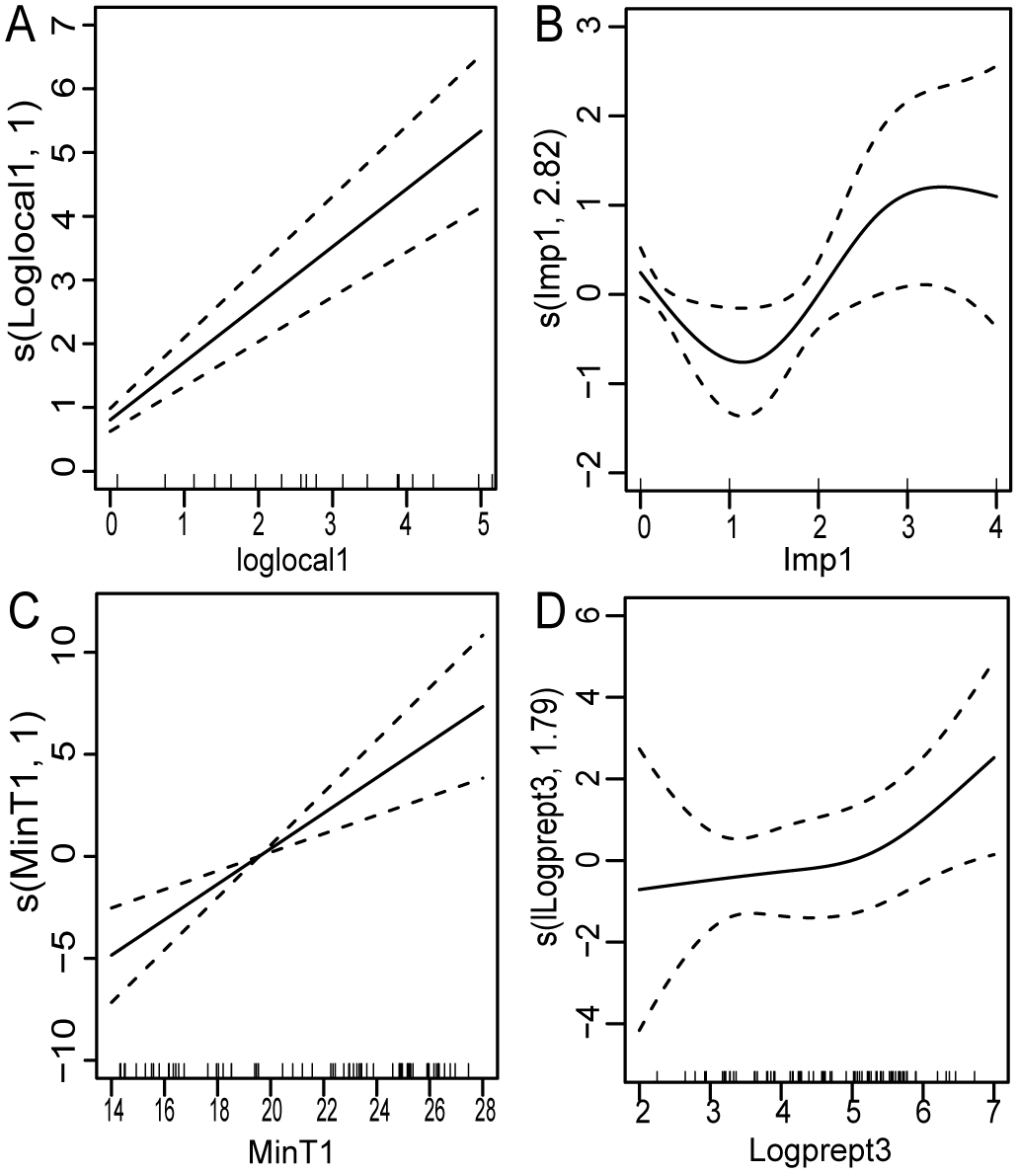
The estimated effects of local cases in the previous month with logarithm transformation (A), imported cases in the previous month (B), minimum temperature in the previous month (C) and cumulative precipitation with three month lags with logarithm transformation (D) on local dengue incidence.

The climatic factors finally included in the model were minimum temperature in the previous month and accumulative precipitation with three month lags (S3 Table) (S4 Table). The time-series Poisson model showed that dengue incidence was positively associated with local dengue incidence in the previous month, imported cases in the previous month, minimum temperature in the previous month, positively associated with accumulative precipitation with three month lags. While the estimated effect of minimum temperature had a linear relationship with the dengue occurrence, the estimated effect of accumulative precipitation was non-linear, more precipitation with three month lags associated with more possibility of dengue occurrence. The estimated effect of imported cases in the previous month was also non-linear for dengue occurrence, the effect of imported cases in number 3 and 4 was larger than imported cases in number 1 and 2 (Fig 3).

**Fig 4:**
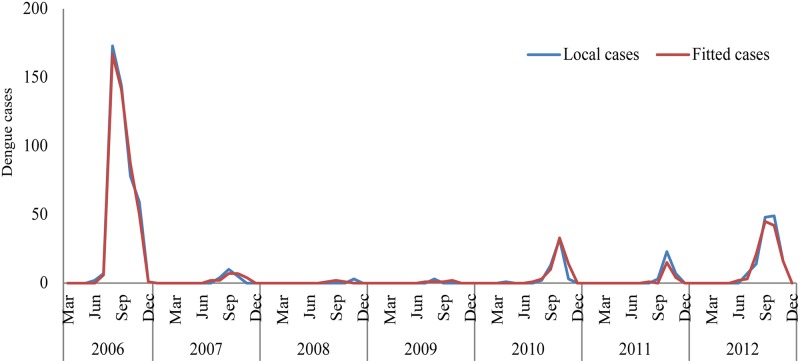
Monthly reported local dengue cases and fitted dengue cases from March 2006 to December 2012.

**Fig 5:**
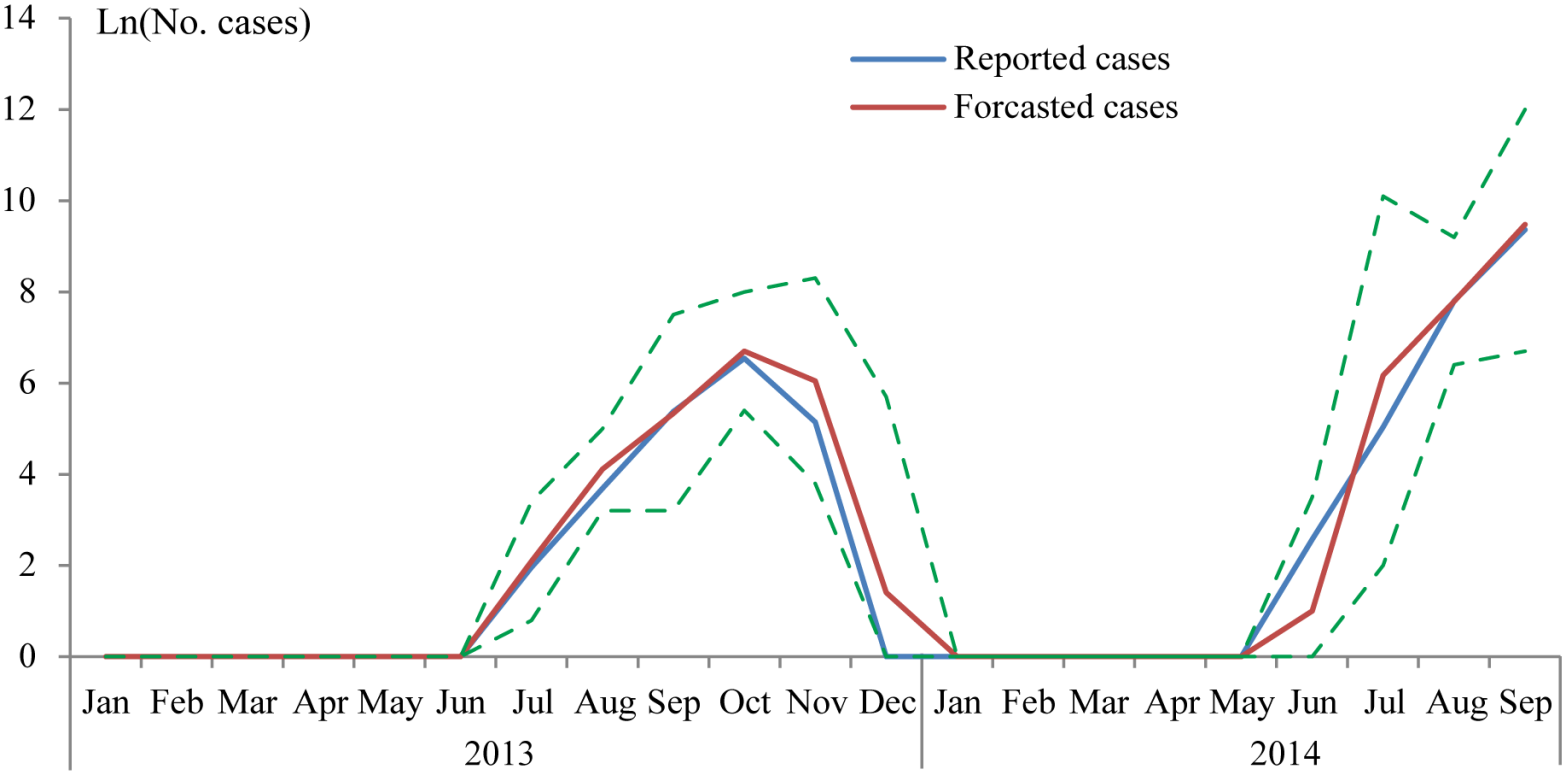
Forecasted dengue cases versus reported local dengue cases from January 2013 to September 2014. The dashed lines represented 95% credible intervals of forecasted dengue cases.

The R^2^ of our model was 0.98, with deviance explained 95.4%. The fitted result shown in Fig 4 exhibited a good fit of the model. The residual test by autocorrelation function (ACF) and partial autocorrelation function (PACF) showed residuals were not correlated (S1 Fig). The forecast result showed that the optimal model could predict the large dengue outbreaks which occurred in 2013 and 2014 (Fig 5).

## Discussion

This study showed that imported cases, minimum temperature and accumulative precipitation could be used to build a low-cost effective dengue early warning model in Guangzhou. Based on these predictors, we may be able to predict large dengue outbreaks one month ahead in Guangzhou and other regions with similar climatic and demographic characteristics, after controlling the autocorrelation, seasonality and long-term trend.

Several studies have been conducted to develop dengue early warning system based on weather factors [16,31,32,33], but these studies were mainly conducted in endemic regions and the transmission vector was *Ae*. *aegypti*. There are two major characteristics of dengue in China: 1) dengue is an imported disease [34]; and 2) the main transmission vector is *Ae*. *albopictus* (the sole transmission vector in Guangzhou). Many travellers to Guangzhou come from Southeast Asia including Indonesia, Philippines, Malaysia, Thailand, and Vietnam. Southeast Asia is an epicentre of dengue and our previous study showed the imported cases in Guangzhou were mainly from Southeast Asia [12]. Furthermore, there is an argument that dengue originated from Africa [35,36], and evidence shows that dengue outbreaks are increasing in size and frequency in Africa [4]. The number of Africans travelling to Guangzhou has been significantly increasing in recent years, which may increase the risk of dengue virus imported to Guangzhou. In fact, a report suggested that the dengue outbreak occurred in Guangzhou was caused by an imported case from Tanzania in 2010 [37]. The imported cases in our study had an increased trend corresponding to dengue occurrence. A study conducted in Taiwan, China demonstrated that imported cases served as initial facilitators for possible dengue transmission and the effect of imported cases could last 14 weeks [38]. The population density increased linearly reaching 6425.4/km^2^ in our study area in 2013. Densely populated conditions provide more susceptible population and suitable *Aedes* mosquito larval habitats [39], which makes the imported virus dispersed more possible.

Endemicity has not been established in some areas for climates that may not support year-round viral transmission. Therefore, local climate also plays a crucial role in dengue transmission besides imported cases in China. *Ae*. *albopictus* is considered to be one of the world’s fastest spreading invasive animal species and has colonized every continent except Antarctica [40], of which temperature plays a crucial role in the population establishment. Temperature also plays an important role in dengue transmission mediated by *Ae*. *albopictus*. Dengue transmission is only achieved when the longevity of the *Ae*. *albopictus* exceeds the EIP. The EIP decreased when temperature increased from 18°C to 31°C, and dengue virus might not be transmitted by *Ae*. *albopictus* at temperatures below 18°C [41]. Furthermore, temperature can also influence the mosquito dynamics by determining their first gonotrophic cycle (FGC), the time between taking a blood meal and first oviposition. Study showed that the length of FGC decreased non-linearly when temperature increased [42]. A greater of proportion of mosquitoes survive the EIP and FGC, thus they are more likely to deliver virus or oviposit a greater number of eggs. Temperature not only impacts the FGC of *Ae*. *albopictus*, but also influences the immature development. *Ae*. *albopictus* developed more quickly at higher temperature within the range of 20–30°C [13,43]. Our results showed that the effects of temperature linearly increased when temperature increased. The decomposition result of minimum temperature showed that the minimum temperature had a decreased trend over the study period, but the minimum temperature was above 18°C from March to October, which fulfilled the possibility of dengue transmission.

*Ae*. *albopictus* can breed in both domestic and peri-domestic containers. The *Ae*. *albopictus* breeding sites in Guangzhou included flower plot trays, bamboo tubes, metal containers, terrariums, stone holes, ceramic vessels, plastic containers, gutters, used tyre dumps, surface accumulated water, and disposable containers [44]. The residents living in the study areas are familiar with raising flowers named evergreen and lucky bamboo cultured with pure water, and they are reluctant to refresh the water for fear of suppressing flower growth. In addition, many residents in these areas enjoy decorating roofs with hanging gardens directly exposed to the outside. The hanging gardens, flowers indoors and garden breeding sites, create the characteristics of spatial distribution of breeding sites. Heavy rain can flushes away the egg, larvae and pupae of *Aedes* mosquitoes in the short term, but rainfall can create huge breeding habitats for mosquito in the long run. Therefore, it is reasonable that more precipitation with three month lags had increased the effect of dengue transmission in current month. A study conducted in Taiwan, China showed that extreme precipitation influenced the dengue occurrence with 70 day lagged effects [45]. Real estate developments and urbanisation increased over these years in Guangzhou, and construction sites created ideal conditions for mosquito breeding. It is reported that dengue outbreaks occasionally occurred at the construction sites in the study areas.

In recent years, dengue became more and more serious in Guangzhou, China and the record of dengue cases in 2014 is unprecedented in nearly 40 years. China has had to face the unprecedented challenges for dengue control and prevention, both currently and into the future. Given there is no effective medication and vaccination, mosquito control is still the only effective way to prevent and control dengue occurrence and outbreak. Although some novel interventions, for example, *wolbachia* [46,47] and genetic modification [48] have made rapid progress in the control of *Ae*. *aegypti*, no achievement has been made in *Ae*. *albopictus*. Therefore, routine interventions on mosquito control, for example breeding sites eradication and eliminating adult mosquito are still the main approaches being used. At population level, it is very important to establish an effective platform to get different stakeholders working together for the disease control and prevention, including government organisations, CDCs and local communities. To establish a dengue early warning system will be an important step in this regard, as it will play a crucial role in dengue control.

A further step, we will develop a user-friendly platform integrating data including dengue cases from China Notifiable Disease Surveillance System, meteorological data from China Meteorological Data Sharing Service System and analysis model. The platform will be configured in Guangzhou CDC and automatically predict the dengue cases occurrences in an upcoming month. The predicted outbreak message will be sent to district CDCs and public health decision-makers for further response. The district CDCs will validate the predicted outbreak and intensify the entomological surveillance. A proposal will then be submitted to local government departments such as Health, Community Service, Emergency management, to advocate and mobilise the community promptly to eradicate mosquito breeding sites and eliminate adult mosquito. The designated organisations will be responsible for public places to manage mosquito density. The platform will be piloted for one or two years. Based on the sensitivity and specification of outbreak predicting, the parameters will be revised and then put into practice on a large scale.

## Supporting Information

S1 Fig Autocorrelation (A) and partial autocorrelation (B) of residuals in Guangzhou. (TIF)

S1 Table The spearman correlation result between local dengue occurrence and climatic factors. (XLSX)

S2 Table The seasonal component of the time series. (XLSX)

S3 Table The significant smoothing predictors included in the model. (XLSX)

S4 Table The top ten models as GCV scores. (XLSX)
