# Supplementary figures and images for: Predicting Unprecedented Dengue Outbreak Using Imported Cases and Climatic Factors in Guangzhou, 2014
Source: PLoS Negl Trop Dis. 2015 May 28;9(5):e0003808. doi: 10.1371/journal.pntd.0003808 (PMC4447292; doi:10.1371/journal.pntd.0003808)

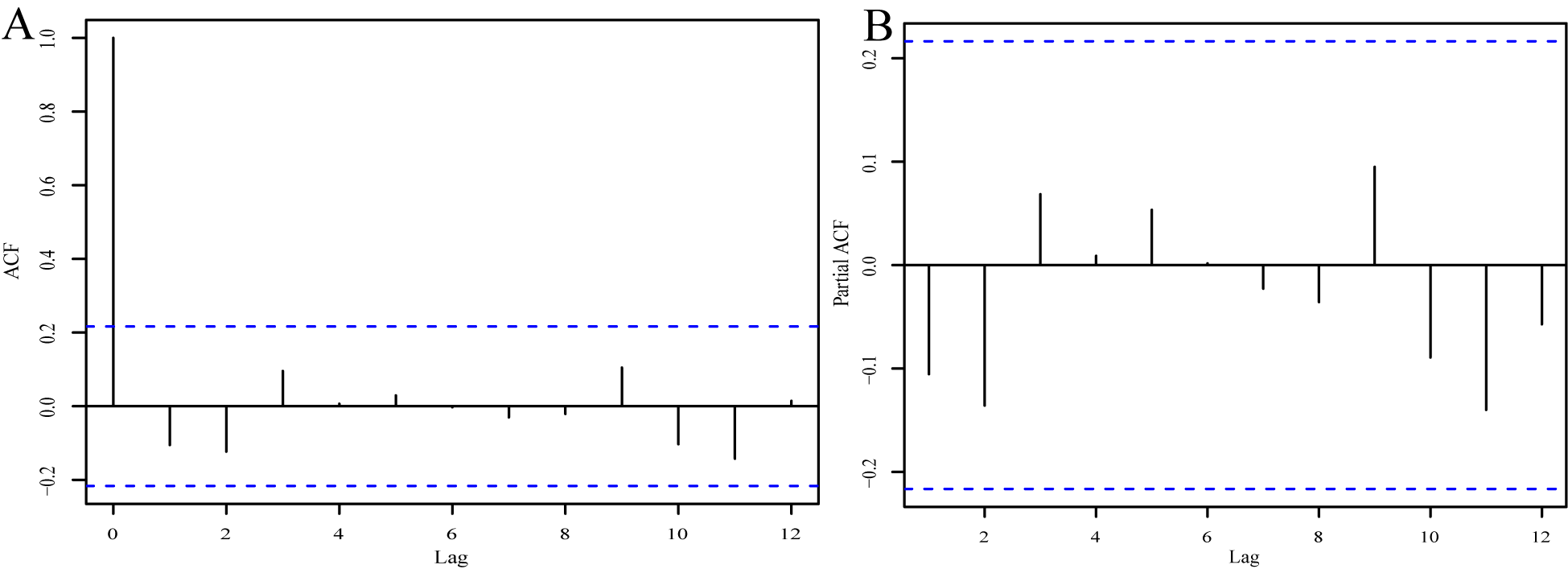

Supplement: S1 Fig — (TIF) [file pntd.0003808.s001.tif]
